# A randomized double blind crossover placebo-controlled clinical trial to assess the effects of a mouthwash containing chlorine dioxide on oral malodor

**DOI:** 10.1186/1745-6215-9-71

**Published:** 2008-12-09

**Authors:** Kayoko Shinada, Masayuki Ueno, Chisato Konishi, Sachiko Takehara, Sayaka Yokoyama, Yoko Kawaguchi

**Affiliations:** 1 Department of Oral Health Promotion, Graduate School, Tokyo Medical and Dental University, 1-5-45 Yushima, Bunkyo-ku, Tokyo 113-8549, Japan

## Abstract

**Background:**

Previous research has shown the oxidizing properties and microbiological efficacies of chlorine dioxide (ClO_2_), however, its clinical efficacies on oral malodor have been evaluated only with organoleptic measurements (OM) or sulphide monitors. No clinical studies have investigated the inhibitory effects of ClO_2 _on volatile sulfur compounds (VSCs) using gas chromatography (GC). The aim of this study was to assess the inhibitory effects of a mouthwash containing ClO_2 _on morning oral malodor using OM and GC.

**Methods:**

A randomized, double blind, crossover, placebo-controlled clinical trial was conducted among 15 healthy male volunteers, who were divided into 2 groups. In the first test phase, the group 1 subjects (N = 8) were instructed to rinse with the experimental mouthwash containing ClO_2_, and those in group 2 (N = 7) to rinse with the placebo mouthwash without ClO_2_. In the second test, phase after a one week washout period, each group used the opposite mouthwash.

Oral malodor was evaluated before rinsing, right after rinsing and every 30 minutes up to 4 hours with OM, and concentrations of hydrogen sulfide (H_2_S), methyl mercaptan (CH_3_SH) and dimethyl sulfide ((CH_3_)_2_S), the main VSCs of human oral malodor, were evaluated with GC.

**Results:**

The baseline oral condition in the subjects in the 2 groups did not differ significantly. The mouthwash containing ClO_2 _improved morning bad breath according to OM and reduced concentrations of H_2_S, CH_3_SH and (CH_3_)_2_S according to GC up to 4 hours after rinsing. OM scores with ClO_2 _were significantly lower than those without ClO_2 _at all examination times. Significant reductions in the concentrations of the three kinds of VSCs measured by GC were also evident at all examination times. The concentrations of the three gases with ClO_2 _were significantly lower than those without ClO_2 _at most examination times.

**Conclusion:**

In this explorative study, ClO_2 _mouthwash was effective at reducing morning malodor for 4 hours when used by healthy subjects.

**Trial registration:**

ClinicalTrials.gov NCT00655772

## Background

Oral malodor, also called halitosis or bad breath, is a general term used to describe an offensive odor emanating from the oral cavity. It is caused by several factors [1]. Although some extraoral causes (nasal inflammation, diabetes mellitus, uremia, etc.) have been suggested, clinical studies have shown that intraoral causes such as gingivitis, periodontitis and tongue coating are the main sources [2-4]. Volatile sulphur compounds (VSCs) (mainly hydrogen sulphide (H_2_S), methyl mercaptan (CH_3_SH) and dimethyl sulphide ((CH_3_)_2_S)), are the major components of malodor originating in the oral cavity. VSCs are mainly produced though putrefactive activities of bacteria residing in the oral cavity. The substrates for VSCs are sulphur-containing amino acids (i.e. cysteine, cystine and methionine) that are found in saliva, gingival cervical fluid and tongue coating debris [5]. To target these microorganisms, and thus to treat oral malodor, different kinds of topical antimicrobial agents have been used.

Antibacterial agents such as chlorhexidine(CHX), cetylpyridinium chloride(CPC), triclosan, essential oils, zinc salts, hydrogen peroxide, sodium bicarbonate and chlorine dioxide(ClO_2_) have been tested, either alone, in different combinations, or together with mechanical devices, for their efficacy to reduce oral malodor [6]. CHX being the most studied antimicrobial agent has also been tested for its efficacy in the treatment of oral malodor. Results from a case series study in halitosis patients suggested a significant effect of 0.2% or 0.12% CHX rinsing [7,8]. More recently, a mouthrinse formulation combining a 0.05% CHX mouthrinse with 0.05% CPC and zinc-lactate was assessed in a double-blind randomized placebo-controlled clinical trial with halitosis patients. The results after 15 days demonstrated its efficacy by significantly reducing in halitosis-related outcome variables [9,10]. Although CHX is considered the most effective oral antiseptic agent, the use of CHX for extended periods of time is related to some side-effects, such as tooth and tongue staining, bad taste and reduced taste sensation [11,12]. Notwithstanding this limitation, mouth rinses containing CHX, CPC and zinc lactate have been demonstrated clinically to be effective, monotherapy, mouth rinses to reduce oral malodor [13].

A proliferation of oral bacteria during sleep is responsible for the release of offending gases, most of which are VSCs, in morning breath even in healthy people [14]. A substantial proportion of healthy people complain of oral malodor. Healthy individuals who suffer from bad breath are likely to use mouthwashes containing several masking or antimicrobial agents. Therefore, recent papers have pointed out the relevance of comparative studies to verify the efficacy of the mouthwashes on morning bad breath in healthy subjects [15-17].

Recently a mouthwash containing chlorine dioxide (ClO_2_) has become available on the Japanese market (ClO2 Fresh^®^, Bio-Cide International, Inc., Oklahoma, USA and Pine Medical co., Tokyo, Japan). Oral rinses containing ClO_2 _are now utilized in dental practices as a topical antiseptic for the oral cavity or for dentures [18-20]. Previous studies have suggested that ClO_2 _and chlorite anion (ClO_2_^-^) directly oxidize VSCs to non-malodorous products and, through this oxidation, consume the amino acids that act as precursors to VSCs [21]. Moreover, chlorite anion is powerfully bactericidal to odorigenic microorganisms [20,22,23].

In most clinical reports, oral malodor was evaluated by organoleptic measurements (OM) or the total concentration of VSCs measured by a portable sulfide monitor (Halimeter™, Interscan co., California, USA) [18,24]. Portable sulfide monitors with a semiconductor gas sensor can detect not only VSCs but also other volatile compounds [25,26]. Because gas chromotography (GC) can quantitatively analyze concentrations of the three main malodor-causing substances (H_2_S), (CH_3_SH) ((CH_3_)_2_S), it is considered one of the most reliable measurements for diagnosing halitosis [27,28]. On the other hand, OM is regarded as the gold standard for evaluating malodor clinically, although it lacks objectivity [29]. OM closely simulates any situations in which malodor is detected [30,31].

Because the effective antimicrobial action of a mouthwash containing ClO_2 _has been verified in vitro [21], the hypothesis tested in this study is that the mouthwash will also effectively reduce oral malodor in humans when VSCs are analyzed with GC. Thus, the aim of this study was to clinically evaluate how well a mouthwash containing ClO_2 _reduces morning oral malodor in healthy subjects using two assessment methods, OM and measuring the concentrations of H_2_S, CH_3_SH and (CH_3_)_2_S with GC. It should be noted that this study is exploratory in design, to test the "clinical potential" of the agent (ClO_2_) rather than its relative effectiveness or broad application across different population groups.

## Materials and methods

### Subjects

Subjects were 15 healthy male volunteers aged 19–38 years (mean age 22.9 ± 6.2 years) who had no medical disorders, were not undergoing antibiotic or other antimicrobial therapy, and were non-smokers. The subjects received verbal and written information about the study and signed consent forms to participate. An oral examination was conducted to assess oral status of the subjects prior to the experiment. We excluded females' subjects because their menstrual cycle might affect oral malodor on the cross over design with one week washout [32].

The sample size was estimated using an expected mean OM score difference of 1, a within-subject variance around the mean OM score difference of 0.5, a significance level of 5%, and a power of 80%. The results indicated a required sample size of 15 subjects for a crossover design.

### Study design

This clinical trial was a randomized, double blind and crossover design with a one week washout period between the crossover phases. The subjects were randomly assigned to 2 groups using a computer-generated random number. In the first test phase, the subjects in one group (N = 8) were instructed to rinse with 10 ml of the experimental mouthwash for 30 seconds while subjects in the other group (N = 7) rinsed with 10 ml of the placebo mouthwash. After the one-week washout period to exclude a carry-over effect of the experimental mouthwash, the second test phase was performed in the same way, but each subject rinsed with the opposite mouthwash. There were no significant differences in any malodor measures between the two groups at either the first or second baseline measurements before mouth rinsing. Similarly, there were no significant differences between the first and second baseline measurements after one week washout period. Hence, we think the oral condition returned to baseline during the one week washout period, and that one week washout is long enough for an explorative trial such as this.

The experimental (with ClO_2_) and the placebo mouthwashes (without ClO_2_) were prepared by Pine Medical Co. for this study. Neither the examiner nor the subject knew whether the mouthwash was experimental or placebo. The contents of each mouthwash were as follows: The experimental mouthwash (ClO_2 _Fresh^®^) contained 0.16% sodium chlorite (NaClO_2_) with an efficacy of 0.1% chlorine dioxide (ClO_2_), glycerin, mint oil, 1.13% citric acid (a pH adjusting agent) and distilled water. The placebo mouthwash contained glycerin, mint oil and distilled water; essentially the same contents as those in the experimental mouthwash except for the ClO_2_. Both mouthwashes were thoroughly membrane filtered and put into plastic bottles sealed with a screw-cap.

### Oral malodor assessment

Measurements were conducted at around 9 o'clock in the morning to evaluate morning breath odor. Subjects were instructed to abstain from eating strong-smelling foods for at least 48 hours, from using scented cosmetics for 24 hours and from drinking alcohol for 18 hours before the assessment. In addition, they were advised not to ingest any food or drink, and to omit their usual oral hygiene practice on the morning of the assessment day [15]. Oral malodor was evaluated before rinsing (baseline), just after rinsing and every 30 minutes thereafter (0, 3, 30, 60, 90, 120, 150, 180, 210, 240 minutes after rinsing) for 4 hours.

#### Organoleptic Measurement

The OM score was measured by two trained judges after subjects closed their mouth for 3 minutes. Judges were asked to rate malodor on a 0–5 score, where a score of 0 represented absence of odor, 1 barely noticeable odor, 2 slight malodor, 3 moderate malodor, 4 strong malodor and 5 severe malodor [33]. If two judges gave different scores a mean score was used as the representative score for the subject. The inter-examiner reliability, using Cohen's kappa test, was 0.72–0.76.

#### Gas chromatography analysis

The GC analysis was carried out using a GC8A gas chromatograph (Shimadzu co., Kyoto, Japan), equipped with a flame photometric detector. After subjects closed their mouth for 3 minutes, the Teflon^® ^tube connected to the auto-injector was inserted into the center of the oral cavity through the lips and teeth, while the lips remained closed. Following aspiration of 20 ml of mouth air with a syringe connected to the outlet of the auto-injector, a 10 ml sample of air was transferred to the column and chromatograph [34]. A sulfur chemiluminescence detector that specifically responds to sulfur was used. VSCs were identified by characteristic retention times and were quantified via comparison of their peak area with that of dilutions of standards. Standard gases of H_2_S, CH_3_SH and (CH_3_) _2_S were prepared with a PD-1B permeater (Gastec co., Kanagawa, Japan). Before the assessment, the ambient air was used for a baseline calibration. Olfactory threshold levels: H_2_S > 1.5 ng/10 ml, CH_3_SH > 0.5 ng/10 ml and (CH_3_) _2_S > 0.2 ng/10 ml, were used according to Tonzetich's criteria [1].

### Statistical Analysis

Statistical analysis was performed using the software program Statistical Package of Social Science (SPSS 15.0J). Means and standard deviations of the clinical indices were calculated, following which the oral examination scores between the two mouthwashes were compared with a Student's t-test. The differences of OM scores and the concentrations of the VSCs between before and after rinsing at each examination point were analyzed with paired t-tests. To detect significant differences of malodor changes between the two mouthwashes, two-factor repeated ANOVA and post-hoc paired t-tests were applied. For all the analyses, a 5% significance level was used.

### Ethical approval and registration

The Ethical Committee for Human Research at Tokyo Medical and Dental University approved this clinical study (No.238). The trial is registered with ClinicalTrials.gov protocol registration system, ID NCT00655772.

## Results

### Characteristics and oral status of subjects

All 15 subjects completed the study. Oral statuses of the subjects were as follows (mean ± S.D.): mean number of decayed teeth from dental caries (DT): 2.7 ± 2.0, missing teeth (MT): 0.5 ± 1.4, filled teeth (FT): 5.6 ± 4.2, DMFT: 8.7 ± 5.2, mean periodontal pocket depth: 2.4 ± 0.5 mm, Gingival index [35]: 1.1 ± 0.7, Plaque index [36]: 1.1 ± 0.6 and resting saliva flow rate: 0.4 ± 0.2 ml/min. There were no statistically significant differences in the oral conditions of the subjects in the 2 groups at the beginning of the study.

### Organoleptic measurement

Changes of means and standard deviations for OM scores are presented in Table 1. At baseline, the mean score in subjects prior to using the experimental mouthwash was 2.1, and subjects prior to using the placebo mouthwash was 1.9. There were no statistically significant differences between the two baselines. With ClO_2_, the mean score fell to 1.2 just after rinsing, following which the mean score gradually rose to 1.5, four hours later. Throughout the study the OM score was below 2, a slight malodor level. Statistically significant improvements in oral malodor compared with before rinsing were evident for up to 4 hours.

**Table 1:** Mean and standard deviation of OM score, H_2_S, CH_3_CH and (CH_3_)_2_S

					time(min.)							
					
			baseline	just after rinsing	30	60	90	120	150	180	210	240
OM score	Exp. (n = 15)	mean (SD)	2.10 (0.51)	1.23** (0.32)	1.20** (0.32)	1.37** (0.40)	1.33** (0.45)	1.40** (0.39)	1.46** (0.32)	1.43** (0.42)	1.47** (0.48)	1.50** (0.42)
	Cont. (n = 15)	mean (SD)	1.87 (0.61)	1.70 (0.53)	1.90 (0.43)	1.90 (0.60)	2.00 (0.57)	2.00 (0.53)	2.11 (0.60)	2.00 (0.50)	2.03 (0.44)	1.93 (0.50)
												
H2S (ng/10ml)	Exp. (n = 15)	mean (SD)	5.31 (4.89)	0.12** (0.28)	0.88** (1.28)	1.62** (2.43)	1.17** (1.11)	1.29** (1.12)	1.18** (0.84)	1.72* (1.39)	1.67** (1.05)	1.84* (1.62)
	Cont. (n = 15)	mean (SD)	4.88 (6.61)	3.70 (7.24)	3.30 (3.83)	3.34 (3.36)	3.74 (3.89)	4.79 (4.67)	6.45 (8.36)	4.99 (4.65)	4.49 (4.65)	6.77 (5.96)
												
CH3SH (ng/10ml)	Exp. (n = 15)	mean (SD)	1.42 (1.48)	0.04** (0.08)	0.14** (0.20)	0.28** (0.31)	0.27** (0.23)	0.27** (0.24)	0.21** (0.17)	0.32* (0.28)	0.30* (0.23)	0.30* (0.28)
	Cont. (n = 15)	mean (SD)	1.21 (1.45)	0.61* (0.81)	0.80 (0.70)	0.94 (0.79)	1.07 (0.93)	1.40 (1.28)	1.78 (1.76)	1.58 (1.49)	1.45 (1.50)	2.03* (1.51)
												
(CH3)2S (ng/10ml)	Exp. (n = 15)	mean (SD)	0.40 (0.27)	0.02** (0.06)	0.05** (0.20)	0.10** (0.16)	0.09** (0.13)	0.12** (0.14)	0.10** (0.12)	0.12** (0.16)	0.13** (0.12)	0.10** (0.12)
	Cont. (n = 15)	mean (SD)	0.33 (0.33)	0.22* (0.21)	0.28 (0.26)	0.29 (0.25)	0.34* (0.30)	0.46 (0.35)	0.52 (0.39)	0.43 (0.40)	0.42 (0.38)	0.53** (0.40)

OM, organoleptic measurement; H_2_S, hydrogen sulfide; CH_3_SH, methyl mercaptan; (CH_3_)_2_S, dimethyl sulfide; Exp., experimental group; Cont., control group; SD, standard deviation. *, p < 0.05; **, p < 0.01 conparision with the baseline (before rinsing) value. Paired t-test was used for statistical analysis.

**Figure 1:**
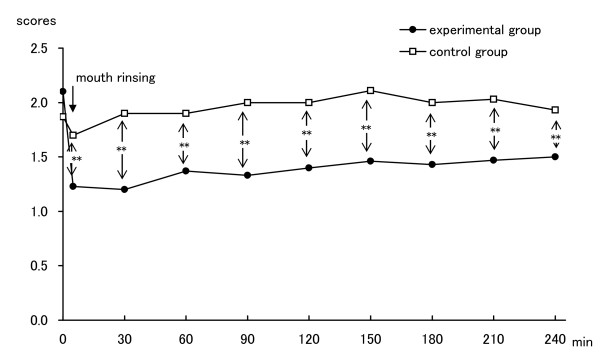
**Changes in organoleptic measurement scores**. Comparison of mean OM values between rinsing with ClO_2 _(n = 15) and rinsing without ClO_2 _(n = 15) by Student's t-test. **, p < 0.01.

Without ClO_2_, on the other hand, the score dropped to 1.7 just after rinsing but it fluctuated thereafter between 1.9 to 2.1. No statistically significant improvement in oral malodor, compared with the score before rinsing, was observed. The scores with ClO_2 _were significantly lower than those without ClO_2 _at all examination times (Figure 1).

### Gas chromatographic assessment

#### Hydrogen sulfide: H_2_S

**Figure 2:**
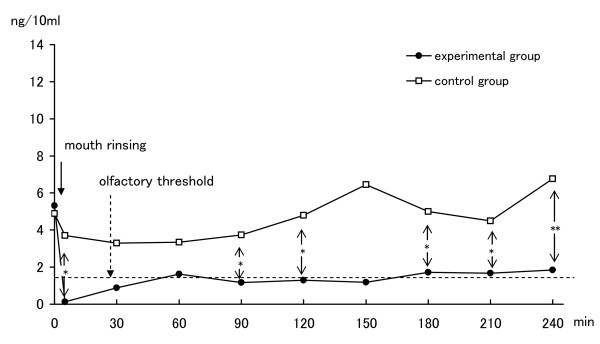
**Changes in concentration of H2S**. Comparison of mean H_2_S, hydrogen sulfide; Olfactory threshold levels, H_2_S > 1.5 ng/10 ml values between rinsing with ClO_2 _(n = 15) and rinsing without ClO_2 _(n = 15) by Student's t-test. *, p < 0.05; **, p < 0.01.

The changes of mean concentrations (ng/10 ml) of H_2_S over the four-hour test period are shown in Table 1. At baseline, the mean concentration in subjects prior to using the experimental mouthwash was 5.31 (ng/10 ml), and prior to using the placebo mouthwash was 4.88. There was no statistically significant difference between the two baselines. With ClO_2_, the mean concentration was reduced to 0.12 just after rinsing. The mean concentration fluctuated but remained below 1.9 for up to 4 hours. These concentrations are below or close to the olfactory threshold levels for H_2_S (1.5 ng/10 ml). Statistically significant improvements compared with before rinsing were evident at all examination times up to 4 hours. Without ClO_2_, however, the change of mean concentrations was trivial. The value fell to 3.70 just after rinsing, a concentration still above the olfactory threshold level. The concentrations with ClO_2 _were significantly lower than those without ClO_2 _at most examination times (Figure 2).

#### Methyl mercaptan: CH_3_SH

**Figure 3:**
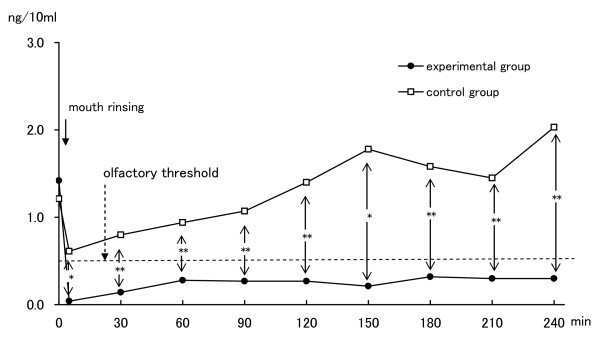
**Changes in concentration of CH3SH**. Comparison of mean CH_3_SH, methyl mercaptan; Olfactory threshold levels, CH_3_SH > 0.5 ng/10 ml values between rinsing with ClO_2 _(n = 15) and rinsing without ClO_2 _(n = 15) by Student's t-test. *, p < 0.05; **, p < 0.01.

The changes of mean concentrations (ng/10 ml) of CH_3_SH over the four-hour test period are shown in Table 1. At baseline, the mean concentration in subjects prior to using the experimental mouthwash was 1.42 (ng/10 ml), and prior to using the placebo mouthwash was 1.21. There was no statistically significant difference between the two baselines. Immediately after rinsing with ClO_2_, the mean concentration declined to 0.04. Concentrations at all examining times after rinsing were much lower than the olfactory threshold level for CH_3_SH (0.5 ng/10 ml). Statistically significant improvements compared with before rinsing were evident at all examination times up to 4 hours. Without ClO_2 _the mean concentration fell to 0.61 (ng/10 ml), however, all measured concentrations were above the olfactory threshold levels for CH_3_SH. The concentrations with ClO_2 _were significantly lower than those without ClO_2 _at most examination times (Figure 3).

#### Dimethyl sulfide: (CH_3_)_2_S

**Figure 4:**
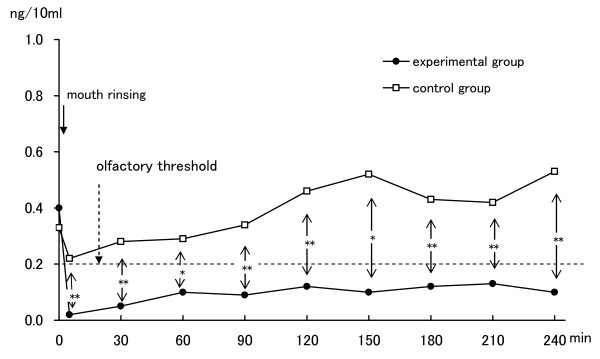
**Changes in concentration of (CH3)2S**. Comparison of mean (CH_3_)_2_S, dimethyl sulfide; Olfactory threshold levels, (CH_3_) _2_S > 0.2 ng/10 ml values between rinsing with ClO_2 _(n = 15) and rinsing without ClO_2 _by Student's t-test. *, p < 0.05; **, p < 0.01.

The changes of mean concentrations (ng/10 ml) for (CH_3_) _2_S over the four-hour test period are shown in Table 1. At baseline, the mean concentration in subjects prior to using the experimental mouthwash was 0.40 (ng/10 ml), and prior to using the placebo mouthwash was 0.33. There was no statistically significant difference between the two baselines. Just after rinsing with ClO_2_, the mean concentration decreased to 0.02, and concentrations remained below the olfactory threshold for (CH_3_) _2_S (0.2 ng/10 ml). Statistically significant improvements compared with before rinsing were evident at all examination times up to 4 hours. Without ClO_2_, the mean concentration dropped slightly to 0.22. The concentration rose gradually thereafter, and all examined concentrations were above the olfactory threshold level. Concentrations with ClO_2 _were significantly lower than those without ClO_2 _at most examination times (Figure 4).

## Discussion

As noted previously, this study design has certain limitations with respect to both generalization from the findings and relative effectiveness in comparison with other agents and the use of a positive control. In this explorative trial, we compared two mouthwashes; one with ClO_2 _and one without ClO_2_, to investigate the malodor reducing effects of ClO_2_. The results of this study demonstrate that rinsing with a mouthwash containing ClO_2 _significantly improves malodor assessed by OM. ClO_2 _mouthwash and also effectively reduces H_2_S, CH_3_SH and (CH_3_)_2_S concentrations in mouth air. Such malodor reducing effects were evident up to 4 hours after rinsing.

Previous studies have suggested that ClO_2 _and ClO_2_^- ^have the ability to directly oxidize VSCs to non-malodorous products and, through this oxidation, consume amino acids such as L-cysteine and L-methionine that act as precursors to VSCs [18,20,23]. Moreover, chlorite anion is powerfully bactericidal to odorigenic microorganisms [18,20,21]. This mouthwash's effect is partially explained by the reduction of the bacterial load in the saliva and on the teeth. The clinical mechanism of oxidization of VSCs and the antibacterial action of ClO_2 _should be confirmed in future studies.

Frascella et al. tested the effectiveness of a ClO_2_-containing mouthwash at different time points for a total of 96 hours after rinsing [37]. The results showed a significant improvement in OM scores and VSCs levels measured by a portable sulfide monitor when the tested mouthwash was compared to a water control at 2 hours after rinsing. The mean VSCs concentration in the test group reached its minimum level at 8 hours after rinsing, but these clinical efficacies on oral malodor were evaluated only with OM or sulphide monitors.

There have been no clinical studies that used GC to investigate inhibitory effects of ClO_2 _on concentrations of H_2_S, CH_3_SH and (CH_3_)_2_S, the main VSCs of human oral malodor. We found that rinsing with ClO_2 _drastically reduced the concentrations of all three, but especially CH_3_SH, far below the olfactory threshold level.

Periodontal disease causes high concentrations of VSCs in mouth air. The concentrations of CH_3_SH are significantly higher in patients with periodontal disease than those in orally healthy individuals [5]. Although the current study was conducted with orally healthy subjects, the results suggest that a mouthwash containing ClO_2 _might lower oral malodor in patients with periodontal disease. However we need to examine the long-term effect as well as the effect on periodontal disease and microbiological consideration of the mouthwash in a future research.

Recently, many over-the-counter mouthwashes have been used in the treatment of oral malodor. Some of these products merely mask malodor. The optimal mouthwash to treat oral malodor would be an antiseptic agent with proven long-lasting efficacy for reduction of OM and VSCs concentrations, with few side effects.

Chlorhexidine-containing mouthwashes inhibit formation of VSCs and are effective oral antiseptics with antiplaque and antigingivitis effects [38]. Although CHX is considered the most effective oral antiseptic agent, rinsing with 0.2% alcohol-free CHX for 1 week caused more irritation to oral mucosa, greater burning sensation, and increased altered taste perception compared to the placebo rinse [11]. Listerine^® ^(Johnson and Johnson, New Jersey, USA), a mouthwash containing essential oils, has clear antiplaque and antigingivitis activity [39]. However, its high alcohol concentration reduces taste sensation and can cause oral pain [40]. Zinc ions inhibit oral malodor but tastes bad [41]. Triclosan and cetylpyridium chloride (CPC) are antimicrobial agents widely used as antiseptic agents [42]. However, their clinical reduction VSCs is questionable [6].

ClO_2 _is used widely in various fields for its safe and high antibacterial action [18,19,23]. Sodium chlorite (NaClO_2_), equivalent to ClO_2_, the traditional ingredient in almost all oxygen supplementation today, is a non-toxic substance approved by the U.S. Food and Drug Administration (FDA) as an antimicrobial agent. We found ClO_2 _not only to be effective at reducing oral malodor, but also none of the volunteers complained about the mouthwash with 0.1% ClO_2 _(0.16% NaClO_2_).

The FDA has established a sodium chlorite level of 0.5% (5,000 ppm) as the maximum allowable concentration for human consumption in food products [43], which is above the level in the experimental mouthwash. Therefore, this mouthwash may have an advantage over other products for oral malodor because of its efficacy and safety.

Most former studies used healthy subjects with no complaints about malodor, lacked an adequate control and evaluated only a short-term effect of up to a few hours. Our study also investigated only short-term effects of the mouthwash and the research design is more like a study model than a clinical trial. Because we only followed our subjects for up to 4 hours our results must be applied to chronic halitosis with caution. It is not known whether the same results would be obtained from halitosis patients. Future research is needed to examine long-term effects, as well as effects on periodontal disease and plaque accumulation in a well-defined sample of halitosis patients. It is also recognized that comparative efficacy studies need to be performed against the known effective mouthrinses containing CHX and that both broader population samples, including females and halitosis patients, should be used in future research. Nonetheless, in this explorative study, the OM score was improved and VSCs concentrations were significantly reduced using the ClO_2 _as an agent. Therefore, the mouthwash clearly demonstrated an anti-malodor effect on morning breath potential without any measurable side effects in healthy subjects.

## Conclusion

The results showed that a mouthwash containing ClO_2 _improved morning bad breath measured with the OM and reduced the concentrations of H_2_S, CH_3_SH and (CH_3_)_2_S measured by gas chromatography for up to 4 hours after mouth rinsing by healthy subjects. However, future studies are needed to examine long-term effects of the mouthwash in halitosis patients. ClO_2 _may have the potential to be included in the range of anti-oral malodor products used to manage this condition.

## Competing interests

The authors declare that they have no competing interests.

## Authors' contributions

KS has made substantial contribution to the study conception and design and obtained the ethics approval. KS, MU, CK, ST and SY implemented this study and participated in the acquisition, analysis and interpretation of data. KS, MU and YK have been intimately involved in drafting and editing the manuscript. All authors read and approved the final manuscript.
